# The CENPN/STAT3/USP37 signaling axis promotes invasion, migration and metastasis in nasopharyngeal carcinoma

**DOI:** 10.3389/fonc.2025.1536574

**Published:** 2025-05-19

**Authors:** Yu-Fei Wang, You Zou, Yue-Long Qiao, Li-Zhi Wu, Shan Xu, Rui Yang, Wo-Er Jiao, Shi-Ming Chen

**Affiliations:** ^1^ Department of Otolaryngology-Head and Neck Surgery, Renmin Hospital of Wuhan University, Wuhan, Hubei, China; ^2^ Swiss Institute of Allergy and Asthma Research (SIAF), University of Zurich, Davos, Switzerland; ^3^ Institute of Otolaryngology-Head and Neck Surgery, Renmin Hospital of Wuhan University, Wuhan, Hubei, China

**Keywords:** transcriptional regulation, nasopharyngeal carcinoma, metastasis, CENPN, STAT3, USP37

## Abstract

**Background:**

Nasopharyngeal carcinoma (NPC) metastasis is the main cause of poor treatment outcomes and death in nasopharyngeal carcinoma patients. Previously, we reported that centromere protein N (CENPN) is closely related to the pathogenesis, radiotherapy resistance and chemotherapy resistance of nasopharyngeal carcinoma, but the relationship between CENPN and nasopharyngeal carcinoma metastasis and its molecular mechanism are still unclear.

**Methods:**

Two nasopharyngeal carcinoma cell lines with stable CENPN knockdown and overexpression were constructed, and changes in their proliferation, invasion and metastasis capacity were detected. Transcriptome sequencing after CENPN knockdown was performed to screen downstream genes regulated by CENPN. The effects of CENPN on the ubiquitin-specific peptide 37 (USP37) transcription were detected via western blotting and qRT-PCR. A bioinformatics analysis revealed that signal transducer and activator of transcription 3 (STAT3) may regulate USP37 transcription. The interaction between CENPN and STAT3 was detected via coimmunoprecipitation, GST pull-down and protein truncation tests. Luciferase reporter, ChIP and mutation assays were used to detect the regulatory effects of STAT3 on USP37 expression. The effect of CENPN on nasopharyngeal carcinoma metastasis *in vivo* was tested in nude mice. The expression of CENPN, STAT3 and USP37 in metastatic tumors from nude mice and human nasopharyngeal carcinoma tissues was verified by immunohistochemistry and immunofluorescence staining.

**Results:**

The invasion and migration capacities of nasopharyngeal carcinoma cells decreased significantly after CENPN knockdown, whereas the overexpression of CENPN significantly promoted the invasion and metastasis abilities of nasopharyngeal carcinoma cells. Transcriptome sequencing showed that USP37 transcription was significantly inhibited after CENPN knockdown, and bioinformatics predicted STAT3 as a potential transcription factor for USP37. Experiments confirmed that CENPN binds directly to STAT3, which regulates USP37 transcription. *In vivo* studies demonstrated a reduced number of liver metastatic tumors in mice injected with CENPN knockdown cells, with decreased expression levels of CENPN, p-STAT3, and USP37. Nonmetastatic NPC tissues also had lower levels of these proteins compared to metastatic tissues.

**Conclusions:**

CENPN directly binds to STAT3 and promotes STAT3 phosphorylation and nuclear translocation to regulate USP37 transcription, thus promoting the invasion and metastasis of nasopharyngeal carcinoma. The CENPN/STAT3/USP37 axis is expected to be a new target for nasopharyngeal carcinoma treatment.

## 1 Introduction

Nasopharyngeal carcinoma (NPC) is a malignant tumor originating from the epithelium of the nasopharynx. It has obvious geographical distribution characteristics, with a high incidence in East Asia and Southeast Asia and a low incidence in other regions (1). In recent years, nasopharyngeal carcinoma has increasingly been recognized as a complex ecological disease characterized by dynamic and multidimensional interactions among various cellular and molecular components. As proposed by Dr. Luo (2), NPC constitutes a pathological ecosystem involving tumor cells, immune cells, stromal elements, and the persistent presence of Epstein-Barr virus (EBV), all interacting within distinct spatial and temporal niches. The onset of nasopharyngeal carcinoma is recessive, and it has a strong tendency to invade and metastasize. Therefore, 6%-8% of nasopharyngeal carcinoma patients already have distant metastasis at the time of presentation (3), and approximately 70% of patients experience cervical lymph node metastasis during the course of the disease (4). Although improvements in radiotherapy techniques and the optimization of chemotherapy strategies in recent years have improved the survival status of nasopharyngeal carcinoma patients (5), the 5-year survival rate is still lower than 20% (6). The main reason is metastasis, the main cause of death in nasopharyngeal carcinoma patients, which has not been resolve (7, 8).

Tumor metastasis is a major challenge in current cancer treatment, and most tumor-related deaths are caused by metastasis. Therefore, a deeper understanding of the molecular mechanism of tumor metastasis is necessary. Cancer cells undergo a series of metastatic cascades to colonize distant organs, in which the epithelial–mesenchymal transformation (EMT) is the initial and key step (9, 10). In this process, EMT-related transcription factors (Snail, Slug, ZEB, Twist, etc.) are expressed; these transcription factors inhibit the expression of E-cadherin, cytokeratins and other epithelial markers and increase the expression of mesenchymal markers, such as N-cadherin, vimentin and fibronectin (11, 12). Cells progressively lose their epithelial characteristics while acquiring mesenchymal phenotypes and thus have a greater capacity to invade and metastasize at distant sites, eventually leading to the spread of cancer cells. Accumulating evidence indicates that neoplastic spindle cells in nasopharyngeal carcinoma exhibit characteristic EMT features, characterized by significant E-cadherin downregulation concurrent with upregulated expression of β-catenin, vimentin, and other mesenchymal markers (13). Notably, this dysregulation of EMT-related biomarkers shows strong clinical associations with unfavorable prognostic outcomes in NPC patients (14).

Centromere protein N (CENPN) is a member of the centromere protein (CENP) family and is essential for centromere assembly. Abnormal expression of the CENP family is an important factor involved in abnormal cell proliferation and division (15). Our previous studies showed that CENPN can promote the malignant biological behaviors of nasopharyngeal carcinoma cells and is closely related to the pathogenesis of nasopharyngeal carcinoma (16). Recent studies have reported that CENPN is associated with the invasion and metastasis of glioma, breast cancer and other cancers (17–20). However, the relationship between CENPN and the invasion and metastasis of nasopharyngeal carcinoma and its molecular mechanism remain unclear.

Signal transducer and activator of transcription 3 (STAT3) is cytoplasmic transcription factor. In normal cells, STAT3 is activated instantaneously through phosphorylation. Then, it transmits transcriptional signals to the nucleus and participates in biological processes such as cell survival, proliferation and differentiation (21, 22). STAT3 plays a well-known role in the process of tumor formation. It is overactivated, abnormally increasing the growth rate and migration ability of tumor cells, resulting in the metastasis of tumor cells and poor prognoses (23, 24). At present, the relationship between STAT3 and the EMT has been clearly established (25), but the regulation of STAT3 and the molecular mechanism through which it affects the EMT remain to be further studied.

The ubiquitin-specific peptide 37 (USP37) enzyme is a deubiquitinase (DUB) that belongs to the family of ubiquitin-specific processing proteases. DUBs are closely related to tumors and play an important role in tumor metastasis (26). Studies have shown that USP37 can directly bind Snail to exert its deubiquitinase activity and regulate the epithelial–mesenchymal transformation (27). Snail (encoded by the SNAI1 gene), an EMT-inducing transcription factor, plays dual roles as both an inhibitor and an activator of transcription, occupying a central position in the regulation of the EMT (28, 29). The Snail protein has poor stability and can be rapidly degraded through the ubiquitination-mediated proteolytic pathway. Through deubiquitination, USP37 increases Snail stability, promotes the EMT, and ultimately promotes the spread and metastasis of tumor cells (30, 31).

In this study, we conducted *in vivo* and *in vitro* experiments to investigate the role of CENPN in regulating the invasion and metastasis capacities of nasopharyngeal carcinoma cells. Furthermore, mechanistic studies revealed that CENPN enhances the transcription of USP37 by promoting the phosphorylation of the transcription factor STAT3. USP37 deubiquitinated the Snail protein to increase its stability, thus promoting the invasion and migration of nasopharyngeal carcinoma cells. The CENPN/STAT3/USP37 axis is expected to provide a new therapeutic target for nasopharyngeal carcinoma metastasis.

## 2 Materials and methods

### 2.1 Bioinformatics analysis and databases

CENPN expression data for head and neck squamous cell carcinoma tissues and control tissues were acquired from The Cancer Genome Atlas (TCGA) database. The Gene Expression Profiling Interactive Analysis tool (GEPIA) was used for the analysis. Four datasets, namely, GSE12452, GSE53819, GSE61218 and GSE118719, from the GEO (Gene Expression Omnibus) database, were selected. R language (R version 4.3.0) was used to analyze the differential expression of CENPN. The survival analysis was performed using the Kaplan–Meier method with the K–M plotter platform. Transcription factors and binding sites were predicted using the GENE database, UCSC database and JASPAR database. See **Supplementary Table S1** for detailed information on the databases.

### 2.2 Patients and specimens

A total of 40 patients with primary nasopharyngeal carcinoma were selected from Renmin Hospital of Wuhan University before February 25, 2023, including 30 patients with metastasis and 10 patients without metastasis. Paraffin-embedded surgical biopsy samples were collected with patient consent. The inclusion criteria were as follows: (1) nasopharyngeal carcinoma confirmed by a pathological examination; (2) no treatment (such as chemoradiotherapy) administered before biopsy; (3) complete imaging evidence to confirm the TNM stage; and (4) patients in the metastatic group had lymph node metastasis or distant metastasis, and patients in the nonmetastatic group had tumors limited to the primary site. This study was approved by the Clinical Research Ethics Committee of Renmin Hospital of Wuhan University (Approval number: 2020K-K221 (Y01)).

### 2.3 RNA sequencing

High-throughput RNA sequencing was performed on CENPN-knockdown NPC cell samples and control samples. DESeq2 software was used to analyze differentially expressed genes (DEGs), with |log_2_FC|≥1 and p<0.05 as the criteria. The sequencing and analysis methods were conducted using previous methods (32).

### 2.4 Immunohistochemistry (IHC)

The paraffin sections were placed in an incubator at 60°C for 20 min, dewaxed in a xylene solution (Sinopharm Chemical Reagent Co., Ltd., Shanghai, China), placed in a gradient of ethanol solutions for rehydration, and then placed in a sodium citrate buffer solution and incubated at 95°C for antigen retrieval. After the solution cooled, the sections were blocked at room temperature with 10% bovine serum albumin (BSA; Sigma-Aldrich, St. Louis, MO, USA) for 1 h. The sections were then incubated at 4°C overnight with the primary antibody. Nonimmune IgG (Cell Signaling Technology, Danvers, MA, USA) was used as a negative control. The sections were incubated at room temperature with secondary antibodies (Servicebio, Wuhan, China) for 1 h in the dark, visualized with diaminobenzidine (DAB; ZSGB-BIO, Beijing, China), and finally restained with hematoxylin (Solarbio, Beijing, China). Images of the staining results were obtained with a microscope (Olympus BX53, Tokyo, Japan) and analyzed using ImageJ software (version 1.53f51; National Institutes of Health, Bethesda, MD, USA). Immunohistochemical staining was evaluated using the IRS (Immunoreactivity Score) system, which integrates staining intensity (graded 0-3) and the percentage of positive cells (graded 0–4), generating a composite score (0–12) that is independent of tumor type or biomarker specificity (33). To ensure methodological rigor, positive controls (NPC specimens with confirmed Ki67 expression) and negative controls (sections processed without primary antibody) were systematically included in all experiments.

### 2.5 Immunofluorescence staining

Before immunofluorescence staining, paraffin sections of the tissues were treated in the same manner as those used for the IHC experiments. The sections were incubated with primary antibodies at 4°C overnight and then with fluorophore-conjugated secondary antibodies (Servicebio, Wuhan, China) at room temperature in the dark for 1 hour. The nuclei were restained with DAPI (Beyotime, Shanghai, China). After being sealed with an anti-fluorescence quenching sealant (Solarbio, Beijing, China), the sections were observed. Images obtained with a fluorescence microscope (Olympus BX53, Tokyo, Japan) were analyzed using ImageJ software (version 1.53f51; National Institutes of Health, Bethesda, MD, USA).

The procedure used for the immunofluorescence staining of the cells was the same as that described above, except that the cells were inoculated on coverslips in advance.

### 2.6 Cell culture

Two nasopharyngeal carcinoma cell lines and one tool cell line were used. The CNE-2Z cell line was purchased from Genechem Shanghai (Shanghai, China; Cat# GCPC0142923). The human highly metastatic nasopharyngeal carcinoma cell line 5-8F was a gift from Southern Medical University (Guangzhou, China). The tool cell line 293T is a human embryonic kidney cell immortalized cell line that was a gift from the College of Life Sciences of Wuhan University (Wuhan, China).

CNE-2Z and 5-8F cells were cultured in RPMI-1640 medium (Gibco, Thermo Fisher Scientific, USA)supplemented with 10% fetal bovine serum (Biological Industries, Kibbutz Beit Haemek, Israel) at 37°C with 5% CO2 and saturated humidity. The 293T cells were cultured in high-glucose Dulbecco’s Modified Eagle Medium (DMEM; Gibco, Thermo Fisher Scientific, USA) supplemented with 10% fetal bovine serum (Biological Industries, Israel), and maintained under the same incubation conditions (37°C, 5% CO_2_, saturated humidity).

### 2.7 Cell transfection

In previous studies, we successfully constructed CNE-2Z and 5-8F cell lines with stable CENPN knockdown and overexpression through RNA interference technology (16, 32). We chose two CENPN shRNA sequences to knock down CENPN expression (CENPN shRNA1: 5’-GCCCTGTTAGACATCATTCAAGAGATGATGTCTAACAGGGC-3’; CENPN shRNA2: 5’-GGAGAATGCAGTCTGGATTCAAGAGAAATCCAGACTGCATTCTCC-3’). Real-time quantitative PCR and western blotting assays revealed that CENPN shRNA2 had a more significant effect on knocking down CENPN expression (16). Therefore, the CENPN shRNA2 sequence was used in this study.

The construction of nasopharyngeal carcinoma cells with CENPN knockdown or overexpression were also performed using the aforementioned method according to our previous work (16, 32).

### 2.8 Colony formation assay

Nasopharyngeal carcinoma cells in the logarithmic growth stage were selected. In each well, 500 cells were inoculated and cultured with complete medium for 14 days, during which the medium was changed every 3 days. The cells were fixed for 30 minutes with 4% paraformaldehyde (Solarbio, Beijing, China) and dyed for 30 minutes with 1% crystal violet solution (Beyotime, Shanghai, China).

### 2.9 EdU assay for cell proliferation

For the EdU staining experiments, EdU was prepared in culture medium at a ratio of 1:1000 (Beyotime, Shanghai, China). Nasopharyngeal carcinoma cells in the logarithmic growth stage were inoculated in six-well plates. They were cultured in complete medium until adhesion and then in EdU medium for 2 hours at 37°C. The cells were fixed with 4% paraformaldehyde (Solarbio, Beijing, China) and incubated at room temperature with Triton X-100 (Sigma-Aldrich, St. Louis, MO, USA) for 10 minutes to increase membrane permeability. Apollo reaction solution was added to the wells and incubated for 30 minutes in the dark. The nuclei were restained with DAPI (Beyotime, Shanghai, China). After the samples were sealed, fluorescence microscopy (Olympus BX53, Tokyo, Japan) was used for counting and analysis.

### 2.10 Wound healing assay

Cells in the logarithmic growth stage were inoculated into a six-well culture plate. When the cells grew fully in the plate, scratches were created using 200 μL pipette tips. PBS (Servicebio, Wuhan, China) was used to remove the suspended cells. After 24 hours of culture in serum-free RPMI 1640 medium (Gibco, Thermo Fisher Scientific, USA), the migration distance was observed under an optical microscope.

### 2.11 Transwell assay

The cells used in the transwell assay were starved in serum-free medium for 8 hours in advance.

For the migration assay, 5×10^4^ cells were mixed in 300 µl of serum-free medium and added to each upper chamber of a transwell culture plate (Corning, Cat# 3422, Corning, NY, USA), and medium with a high concentration of serum was added to the lower chamber. After 48 hours of culture, the chamber was removed, and the cells that had not penetrated the upper chamber were removed. The cells were fixed with 4% neutral formaldehyde (Solarbio, Beijing, China) for 15 minutes. After staining with 1% crystal violet (Beyotime, Shanghai, China), the cells that migrated through the membrane were counted using an optical microscope (Olympus CX23, Tokyo, Japan).

For the invasion assay, 1×10^5^ cells were mixed in 300 µl of serum-free medium and added to each upper chamber, which was covered with Matrigel matrix gel (Corning, NY, USA). The other steps were the same as those in the migration assay.

### 2.12 Phalloidin staining assay

The cells were inoculated onto coverslips in six-well plates. After cell adhesion, the medium was discarded, and the cells were fixed with 4% neutral formaldehyde (Solarbio, Beijing, China). The cells were then stained with rhodamine-conjugated phalloidin (Yeasen, Shanghai, China) for 30 min, and the nuclei were restained with DAPI (Beyotime, Shanghai, China). Fluorescence microscopy (Olympus BX53, Tokyo, Japan) was used to acquire images.

### 2.13 Protein extraction and western blotting

The entire process of protein extraction was performed on ice. Culture dishes filled with cells were washed twice with a precooled PBS solution (Servicebio, Wuhan, China). After RIPA lysis buffer (Beyotime, Shanghai, China) and protease inhibitors (Servicebio, Wuhan, China) were added, the cells were gently scraped off with a cell scraper and collected in a centrifuge tube. Ultrasonication was used to disrupt the cell membranes, and the supernatant was collected after centrifugation. The protein concentration was determined using the BCA method (Beyotime, Shanghai, China). Before conducting western blotting assays, the samples were boiled to denature the proteins.

Protein samples were separated on 10% SDS–PAGE gels (Servicebio, Wuhan, China) and then transferred to a 0.45 μm PVDF membrane (Millipore, MA, USA). The membrane was incubated with 5% milk (Beyotime, Shanghai, China) at room temperature for 1 h to block the nonspecific antibody binding sites and then incubated overnight at 4°C with the diluted primary antibody and at room temperature for 1 h with the secondary antibody. Blotting images were obtained with a ChemiDoc imager (Bio-Rad, CA, USA) using ECL solution (Beyotime, Shandong, China). Image analysis was performed using ImageLab (version 5.1, Bio-Rad) and ImageJ software.

The antibodies used are shown in **Supplementary Table S2**.

### 2.14 Nude mouse model of metastatic tumor dissemination

The animal experimental ethics application was approved by Renmin Hospital of Wuhan University (Approval number: WDRM20200815). Four-week-old BALB/c nude mice were purchased from Mouse Noble Biotechnology (Wuhan, Hubei, China) and raised in an SPF barrier environment at the Animal Experimental Center of Renmin Hospital of Wuhan University; 8 mice in the CENPN-knockdown group and 8 in the control group were used. A nasopharyngeal carcinoma metastasis model was established by injecting a tumor cell suspension into the tail vein. A total of 2×10^6^ cells were resuspended in 200 μl of a precooled PBS solution (Servicebio, Wuhan, China) and injected into the tail vein of each nude mouse at a slow and uniform rate. The mice were sacrificed after 4 weeks, and metastatic tumor nodules were counted and averaged by two observers (30).

### 2.15 Protein truncation assay

A protein truncation assay was used to detect the binding fragments of CENPN and STAT3. The truncated amino acid sequence was constructed in the pcDNA3.1 carrier according to the required labels of the truncated amino acid sequence. 293T cells were transfected with plasmids, and protein was extracted and detected via western blotting.

### 2.16 ChIP assay

A ChIP assay was used to determine whether STAT3 can bind to the USP37 promoter region and to verify the binding site of STAT3 in the USP37 promoter. The method was performed as described in the literature (34). The precipitated DNA was detected via real-time quantitative PCR using the following primers: P1, 5’-TCCTCCAAAAGCCAGTGAACG-3’ (forward) and 5’-CTGTAGTAGGCACGCGCA-3’ (reverse); P2, 5’-CTAGTCCCCGCAACTTCTGG-3’ (forward) and 5’-TGTCCATCTTTTCCCGCCG-3’ (reverse); P3, 5’-TTAGGAGAGAAGGCCGAAACG-3’ (forward) and 5’-GCTACGGCGGCTCATTGTTT-3’ (reverse); and P4, 5’-CAAAGAACGCGATGGGTCG-3’ (forward) and 5’-TCTCCTAATTCCCGGTGTCG-3’ (reverse).

### 2.17 Luciferase reporter assay

The pLVX-STAT3-Flag overexpression vector was constructed by inserting the STAT3-Flag CDS region into the lentivirus skeleton vector at the multiple cloning site. The first 1500 bases of the USP37 promoter were inserted into the pGL3 vector (Promega, Madison, WI, USA) to construct USP37-PGL3. Forty-eight hours after 293T cells were transfected, the cells were harvested and treated with the Luciferase Assay System (Promega). The GloMax^®^ 20/20 Luminometer System (Promega, Madison, WI, USA) was used to detect the luminescence signal.

### 2.18 Immunofluorescence colocalization assay

The nasopharyngeal carcinoma cells inoculated on coverslips were fixed, incubated with anti-CENPN and anti-pSTAT3 primary antibodies at 4°C overnight, and then incubated with Cy3-labeled and AlexaFluor488-labeled fluorescent secondary antibodies (Servicebio, Wuhan, China) against the corresponding species at room temperature for 1 hour. After the cell nuclei were stained with DAPI (Beyotime, Shanghai, China), the images were observed with a fluorescence microscope (Olympus BX53, Tokyo, Japan).

### 2.19 Statistical analysis

All experiments included at least 3 biological replicates, and the results are presented as the means ± SDs. Statistical analysis was performed using t tests with GraphPad Prism 8.0.2 (GraphPad Software, San Diego, CA, USA) and SPSS 25.0 (IBM Corp., Armonk, NY, USA). The significance was marked as * p<0.05, ** p<0.01, *** p<0.001 and **** p<0.0001. n.s.: not significant.

## 3 Results

### 3.1 CENPN is strongly associated with nasopharyngeal carcinoma cell proliferation

**Figure 1:**
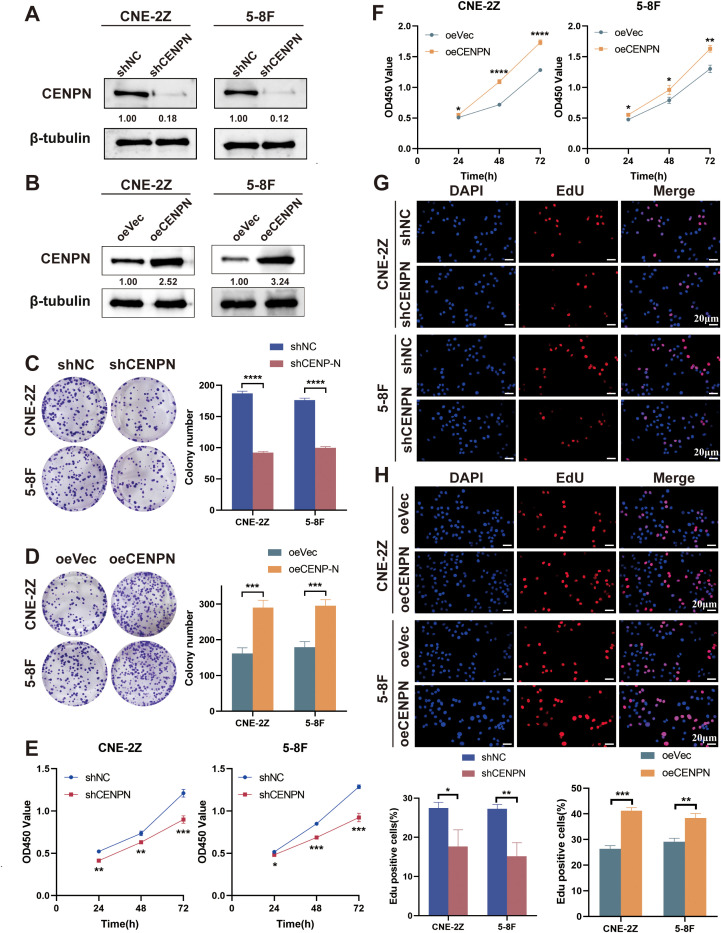
CENPN is related to the proliferation of nasopharyngeal carcinoma cells. **(A, B)** CENPN knockdown and overexpression in CNE-2Z and 5-8F cells were verified by western blotting. **(C, D)** Changes in the colony formation ability of the two cell lines after CENPN knockdown or overexpression. **(E, F)** CCK8 assays of the two cell lines after CENPN knockdown or overexpression. **(G, H)** EdU assays of the two cell lines after CENPN knockdown or overexpression (400×). * p<0.05, ** p<0.01, *** p<0.001 and **** p<0.0001.

We infected the NPC cell lines CNE-2Z and 5-8F with lentiviruses to generate stable CENPN knockdown (shCENPN) and overexpression (oeCENPN) cells, along with their respective control shNC and oeVec cells. Compared with the control group, CENPN expression significantly decreased in the shCENPN groups and increased in the oeCENPN groups for both CNE-2Z and 5-8F cells (**Figures 1A, B**).

Next, we studied the relationships between the expression of CENPN and the clonogenic and proliferative abilities of nasopharyngeal carcinoma cells. Compared with the control group, CENPN knockdown reduced colony formation in CNE-2Z and 5-8F cells, while its overexpression enhanced this ability (**Figures 1C, D**). The results of the CCK8 assay measured at 450 nm and the percentage of EdU-positive cells revealed that CENPN expression significantly promoted the proliferation of nasopharyngeal carcinoma cells (**Figures 1E–H**).

### 3.2 Knockdown of CENPN represses the epithelial–mesenchymal transition, migration and invasion of NPC cells *in vitro*

**Figure 2:**
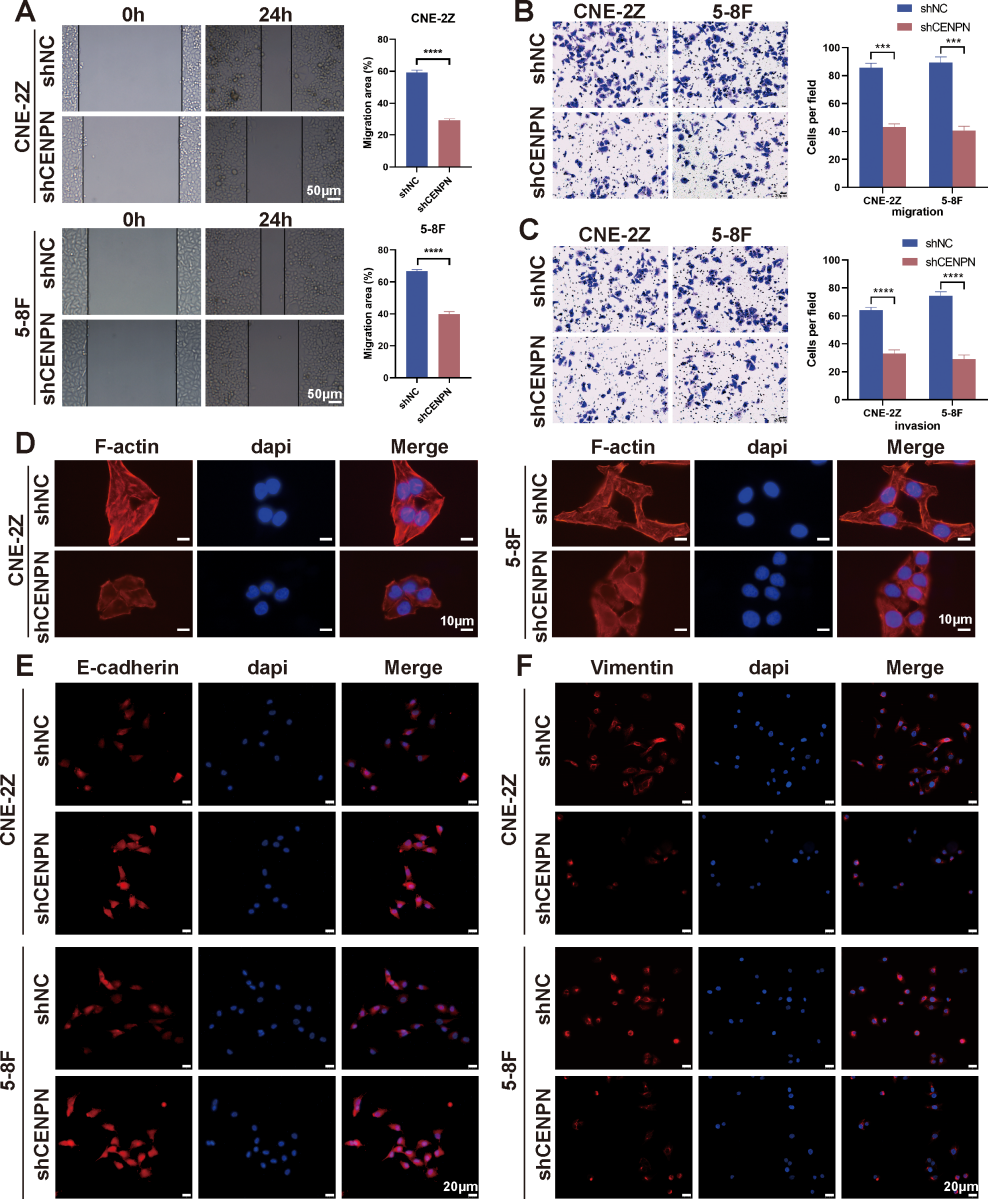
CENPN knockdown inhibits the invasion and metastasis ability of nasopharyngeal carcinoma cells. **(A)** Wound healing assays of the 5-8F and CNE-2Z cell lines after CENPN knockdown (200×). **(B, C)** Transwell assays of the two cell lines after CENPN knockdown (400×). **(D)** Phalloidin staining showing cell morphology changes after CENPN knockdown (1000×). **(E, F)** Immunofluorescence staining of EMT-related proteins after CENPN knockdown (400×). *** p<0.001 and **** p<0.0001.

Given the poor prognosis caused by nasopharyngeal carcinoma metastasis, we focused on the relationship between CENPN expression and metastasis-related biological behaviors. First, we investigated the effects of CENPN knockdown on nasopharyngeal carcinoma cells and found that, compared with the control cells, the CNE-2Z and 5-8F cell lines presented significant decreases in invasion and migration after CENPN knockdown (**Figures 2A–C**). Phalloidin staining revealed the cytoskeletal morphology after CENPN knockdown, and the results showed that the pebble-like epithelial appearance of the cells in the shCENPN group was more obvious than that in the control group (**Figure 2D**). Immunofluorescence showed increased E-cadherin and decreased vimentin expression, indicating enhanced epithelial characteristics in both cell lines (**Figures 2E, F**). For the statistical analysis, see the **Supplementary Figures** (**Supplementary Figure S1**).

### 3.3 Overexpression of CENPN promotes the epithelial–mesenchymal transition, migration and invasion of NPC cells *in vitro*

**Figure 3:**
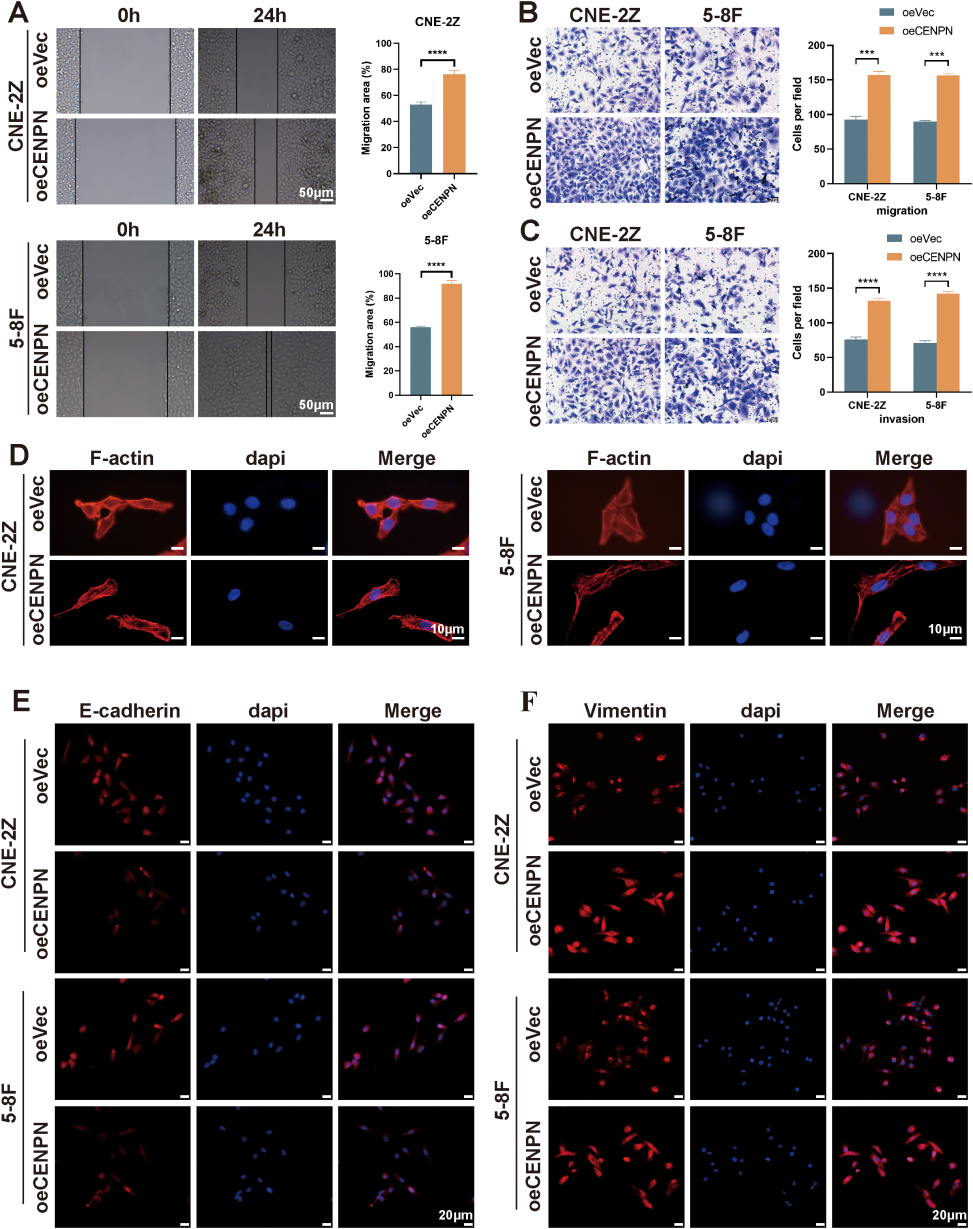
Overexpression of CENPN enhances the invasion and metastasis abilities of nasopharyngeal carcinoma cells. **(A)** Wound healing assays of the 5-8F and CNE-2Z cell lines after CENPN overexpression (200×). **(B, C)** Transwell assays after CENPN overexpression (400×). **(D)** Phalloidin staining showing cell morphology changes after CENPN overexpression (1000×). **(E, F)** Immunofluorescence staining of EMT-related proteins after CENPN overexpression (400×). *** p<0.001 and **** p<0.0001.

We used oeCENPN cells to further verify the effect of CENPN expression on promoting the invasion and metastasis of nasopharyngeal carcinoma cells. Compared with those of the control cells, the invasion and migration abilities of the two cell lines were significantly increased after CENPN overexpression (**Figures 3A–C**). The cell morphology was more elongated, showing a more obvious fusiform mesenchymal appearance (**Figure 3D**). The expression of the epithelial indicator E-cadherin was decreased, while the expression of the mesenchymal indicator vimentin was increased, and the characteristics of mesenchymal cells were more obvious (**Figures 3E, F**). For the statistical analysis, see the **Supplementary Figures** (**Supplementary Figure S2**).

### 3.4 CENPN knockdown suppresses the epithelial–mesenchymal transition, migration and invasion of NPC cells by reducing USP37 expression

**Figure 4:**
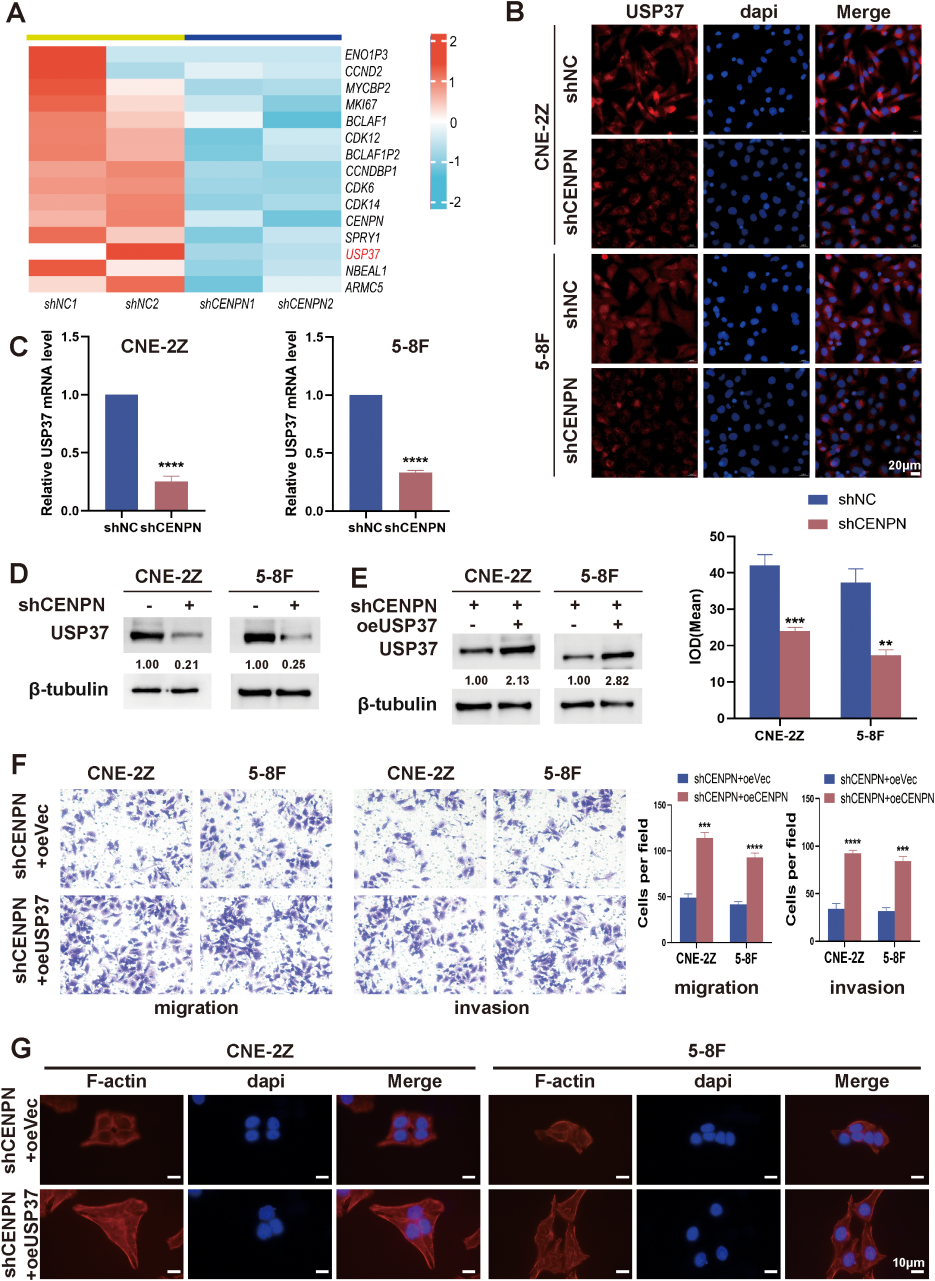
CENPN promotes nasopharyngeal carcinoma cell invasion and metastasis by regulating USP37 expression. **(A)** Transcriptome sequencing revealed downregulated genes after CENPN knockdown. **(B)** Immunofluorescence staining of USP37 after CENPN knockdown (400×). **(C)** qPCR revealed decreased USP37 transcript levels after CENPN knockdown. **(D)** Western blotting detected reduced USP37 protein expression after CENPN knockdown. **(E)** Western blotting confirmed successful establishment of shCENPN+oeUSP37 cells. **(F)** Transwell assays of the shCENPN+oeUSP37 cells. **(G)** Phalloidin staining revealed cell morphology changes (1000×). ** p<0.01, *** p<0.001 and **** p<0.0001.

Transcriptomic sequencing of shCENPN and shNC nasopharyngeal carcinoma cells revealed that USP37 expression was significantly reduced after CENPN knockdown (**Figure 4A**). Moreover, studies have shown that USP37 is closely related to tumor metastasis (30, 31). Immunofluorescence staining, qRT-PCR and western blotting assays confirmed that in the CNE-2Z and 5-8F cell lines, CENPN knockdown significantly decreased the expression of USP37 at both the transcript and protein levels (**Figures 4B–D**).

In CNE-2Z and 5-8F cells, lentiviral transduction was used to construct sequential CENPN knockdown and USP37 overexpression cells (shCENPN+oeUSP37) and the corresponding control group shCENPN+oeVec. The western blotting results confirmed successful construction (**Figure 4E**). Compared with those of the shCENPN+oeVec group, the invasion and migration abilities of the shCENPN+oeUSP37 group were significantly increased (**Figure 4F**), and the cell morphology of the shCENPN+oeUSP37 group was more similar to that of normal nasopharyngeal carcinoma cells (**Figure 4G**). These results indicate that overexpression of USP37 in shCENPN nasopharyngeal carcinoma cells partially compensates for the inhibitory effect of CENPN knockdown on the epithelial–mesenchymal transformation, invasion and migration. CENPN is involved in the invasion and metastasis of nasopharyngeal carcinoma cells via USP37.

### 3.5 CENPN is involved in NPC cell invasion and metastasis by regulating STAT3-USP37 expression

**Figure 5:**
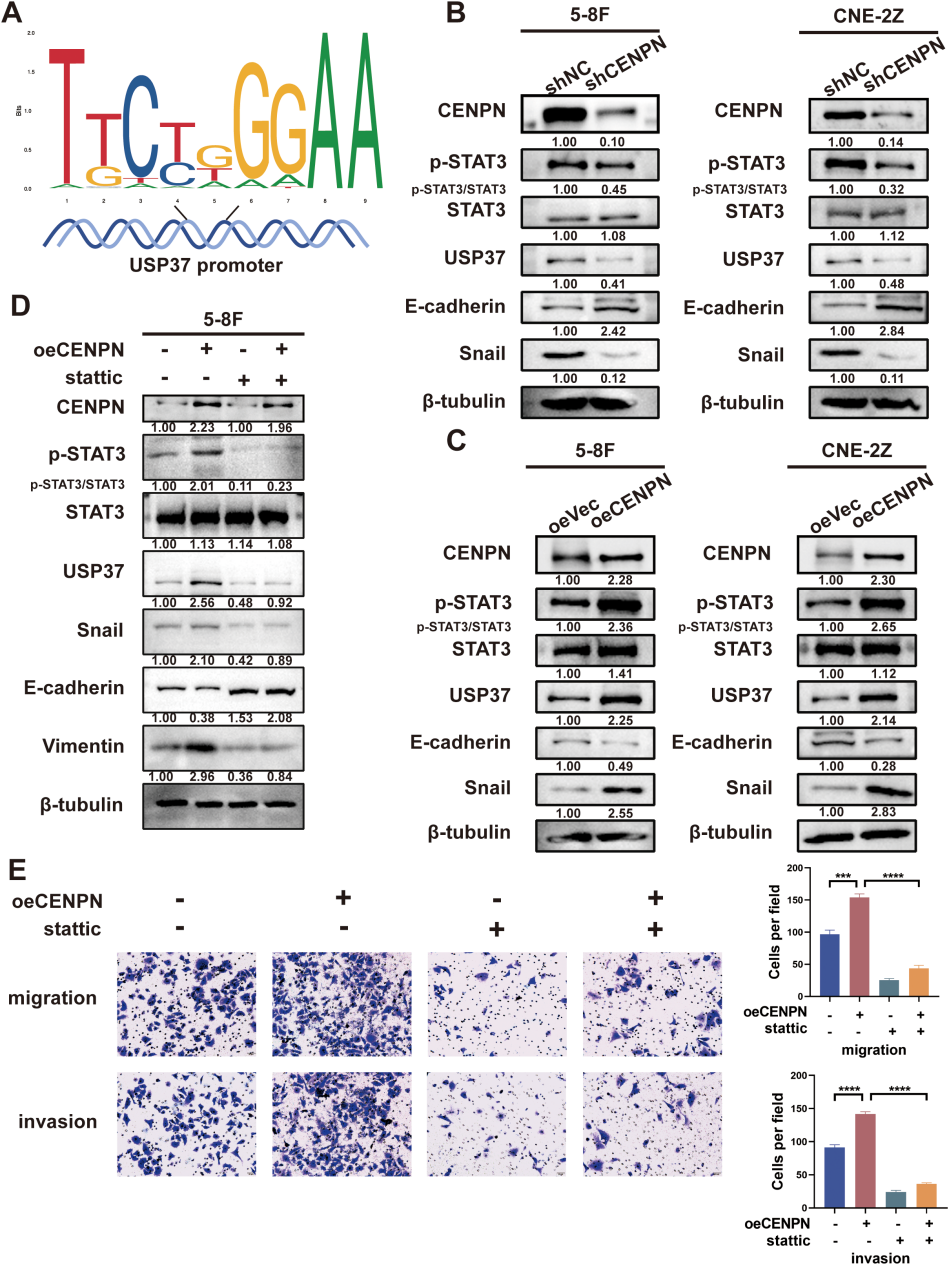
CENPN regulates nasopharyngeal carcinoma cell invasion and metastasis by activating STAT3 to modulate USP37 expression. **(A)** Sequence diagram illustrating the domain of STAT3 binding to the USP37 promoter. **(B)** Changes in p-STAT3 and EMT-related protein expression in the 5-8F and CNE-2Z cell lines after CENPN knockdown. **(C)** Changes in p-STAT3 and EMT-related protein expression in the two cell lines after CENPN overexpression. **(D)** Western blotting showed changes of protein expression in 5-8F cells treated with stattic. **(E)** Transwell assays of 5-8F cells treated with stattic. *** p<0.001 and **** p<0.0001.

Although the above results confirmed that CENPN regulates the metastasis of nasopharyngeal carcinoma cells through USP37, CENPN, as a centromere protein, cannot directly regulate USP37 expression. According to the transcription factor prediction, STAT3 may act as a transcription factor to regulate USP37 expression, and STAT3 binding sites are present in the promoter region of USP37 (**Figure 5A**).

We further investigated whether CENPN regulated STAT3 protein expression. Western blotting results showed that compared with the control group, the expression of p-STAT3, USP37 and Snail in the shCENPN groups of 5-8F and CNE-2Z cells was significantly downregulated, and the expression of E-cadherin was increased (**Figure 5B**). The opposite results were obtained for each protein in the overexpression group (**Figure 5C**). However, no significant change in the expression of STAT3 was detected in any of the groups. These results indicate that CENPN regulates the phosphorylation and activation of STAT3 but has no significant effect on the expression of STAT3.

We treated the oeCENPN group with STAT3 inhibitor stattic to inhibit the activation and nuclear translocation of STAT3 and further determine the role of STAT3 activation in the regulation of USP37 expression by CENPN. Compared with those in the oeCENPN group, western blottings revealed significantly decreased expression of p-STAT3, USP37 and Snail, and increased expression of E-cadherin after stattic addition, whereas the expression of STAT3 did not change significantly (**Figure 5D**). Transwell assays confirmed that stattic significantly inhibited the effect of CENPN overexpression on promoting the invasion and metastasis of nasopharyngeal carcinoma cells (**Figure 5E**). These results suggest that CENPN regulates USP37 expression by promoting the phosphorylation of STAT3, thus regulating the invasion and metastasis of nasopharyngeal carcinoma cells.

### 3.6 Direct binding of CENPN and STAT3 in the cytoplasm promotes STAT3 phosphorylation and its translocation to the nucleus, where it binds specifically to the USP37 promoter

**Figure 6:**
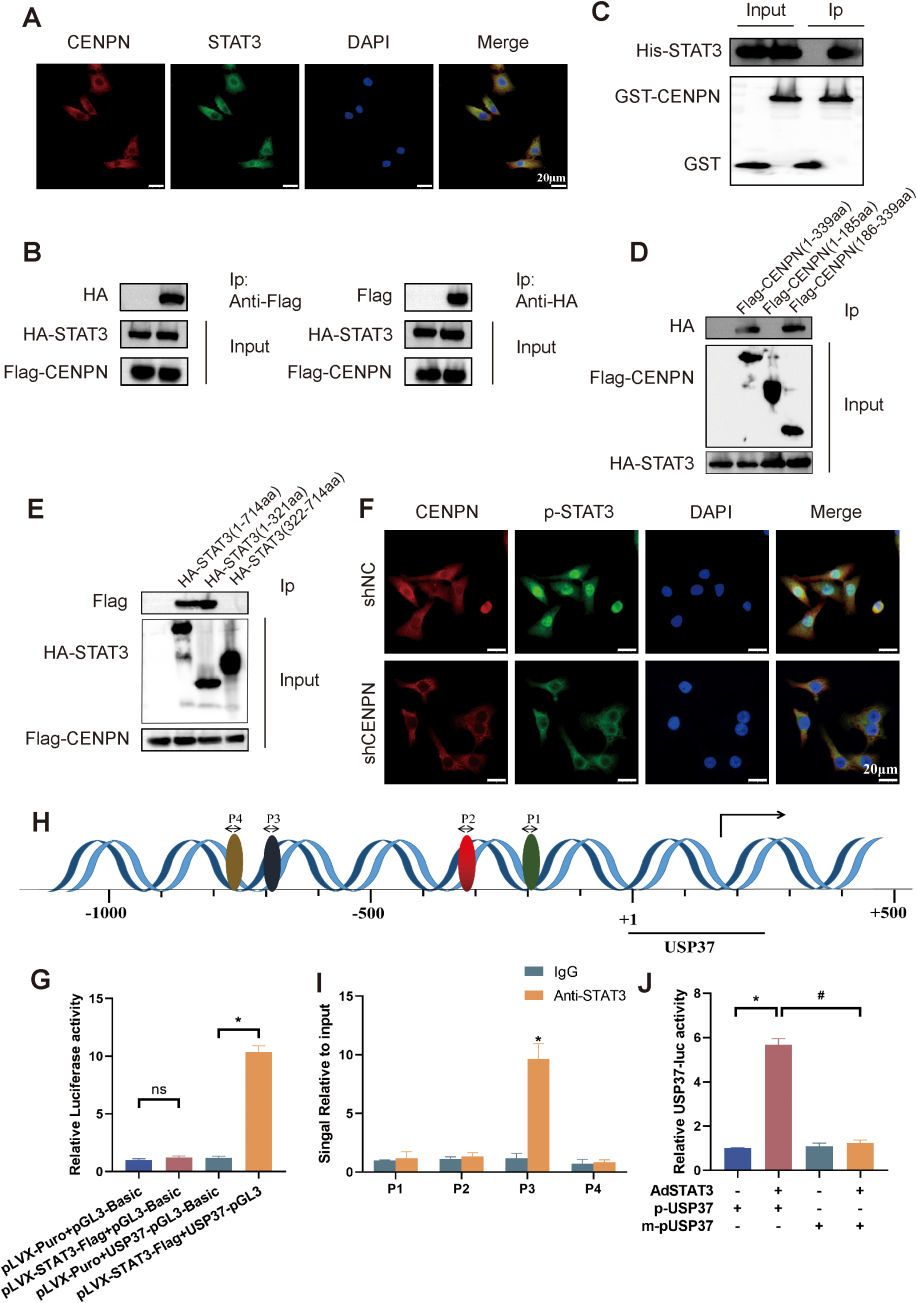
CENPN binds to STAT3 to promote its phosphorylation and nuclear translocation, followed by the specific binding of STAT3 to the USP37 promoter region. **(A)** Immunofluorescence staining showing colocalization of CENPN with p-STAT3 (400×). **(B)** Coimmunoprecipitation demonstrating the interaction between CENPN and STAT3. **(C)** GST pull-down assay showing direct CENPN-STAT3 interaction. **(D, E)** Immunoprecipitation of CENPN and STAT3 fragments after the truncation of protein. **(F)** Immunofluorescence experiment showing the distribution of p-STAT3 in cells (400×). **(G)** Luciferase reporter assay showing that STAT3 binds to the USP37 promoter. **(H)** Bioinformatics prediction of potential STAT3 binding sites in the USP37 promoter. * p<0.05, and ns: not significant. **(I)** ChIP–PCR confirming STAT3 binds to the USP37 promoter at site P3. **(J)** Relative USP37 luciferase activity in 293T cells transfected with AdSTAT3, p-USP37-luciferase, or m-pUSP37-luciferase (with the STAT3 binding site deleted). * p<0.05 vs the pUSP37 group. # p<0.05 vs the m-pUSP37 with AdSTAT3 group.

We first performed immunofluorescence staining of 5-8F cells to explore the detailed molecular mechanism by which CENPN promotes nasopharyngeal carcinoma metastasis, and the results revealed that CENPN and STAT3 were colocalized (**Figure 6A**). Further coimmunoprecipitation assays confirmed the interaction between CENPN and STAT3 (**Figure 6B**). GST pull-down experiments also confirmed their direct interaction (**Figure 6C**). The results of protein truncation assays indicated that the interaction site was the amino acid fragment 186–339 aa of the CENPN protein and the fragment 1–321 aa of STAT3 (**Figures 6D, E**). Immunofluorescence experiments revealed that the distribution of p-STAT3 in the nucleus was significantly reduced after CENPN knockdown (**Figure 6F**). Taken together, these results confirmed that CENPN promoted the phosphorylation and nuclear translocation of STAT3 by directly binding to STAT3.

Next, we conducted a luciferase reporter assay to explore the binding of STAT3 to the USP37 promoter region. The results showed higher relative luciferase activity in 293T cells cotransfected with pLVX-STAT3-Flag+USP37-pGL3 (**Figure 6G**). Bioinformatics predictions revealed four possible binding sites for STAT3 in the USP37 promoter, and chromatin coprecipitation results subsequently confirmed that STAT3 could bind to P3 (−699 to −689) in the USP37 promoter (**Figures 6H, I**). Luciferase reporter assays revealed that the relative USP37 luciferase activity in the AdSTAT3+p-USP37 group was significantly higher than that in the p-USP37, m-pUSP37 and AdSTAT3+m-pUSP37 groups (**Figure 6J**). Therefore, STAT3 can bind specifically to the USP37 promoter region.

These results indicate that CENPN promotes STAT3 phosphorylation and nuclear translocation through a direct interaction with STAT3, thereby promoting the transcriptional activation of USP37 expression and the development of the EMT, ultimately leading to the invasion and metastasis of nasopharyngeal carcinoma.

### 3.7 Knockdown of CENPN represses the distant metastasis of NPC *in vivo*

**Figure 7:**
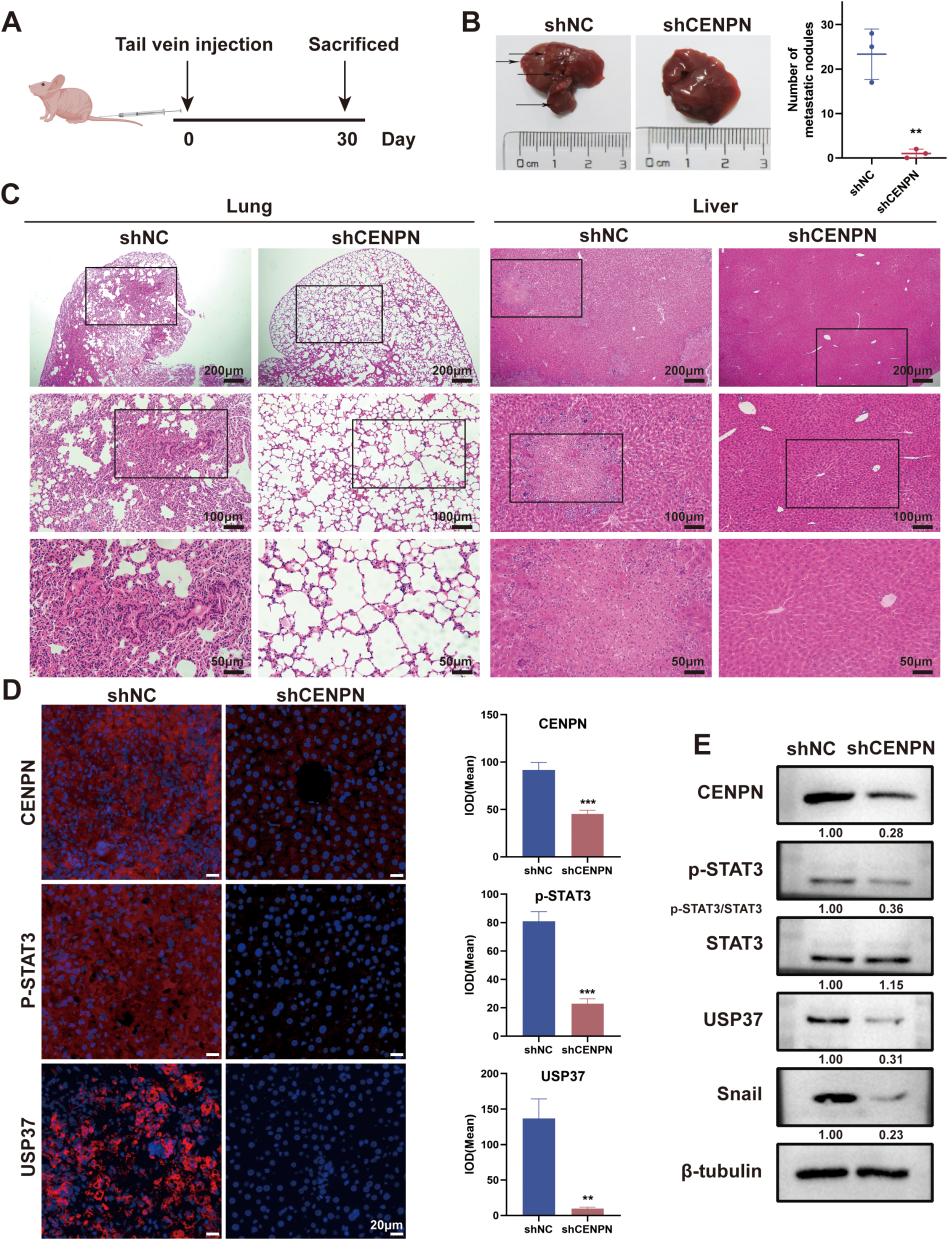
CENPN knockdown inhibits nasopharyngeal carcinoma metastasis *in vivo*. **(A)** Establishment of a nude mouse metastasis model. **(B)** Liver images of nude mice from control and shCENPN groups. **(C)** HE staining of lung and liver sections from the control and shCENPN groups (40×, 100× and 200×). **(D)** Immunofluorescence staining of liver sections from the control and shCENPN groups (400×). **(E)** Western blotting analysis of liver homogenates. ** p<0.01 and *** p<0.001.

We established a nude mouse model of metastatic tumors by injecting nasopharyngeal carcinoma cells through the tail vein to investigate the effect of CENPN expression on nasopharyngeal carcinoma metastasis *in vivo* (**Figure 7A**). Compared with that in the control group, the number of liver metastases and lung metastases in the experimental group injected with shCENPN cells was significantly reduced (**Figure 7B**). HE staining of the livers and lungs revealed that the livers and lungs of the control group lost their normal structure and exhibited obvious cancer nest formation. In the control group, the nucleus–to–cytoplasm ratio in the liver and lung metastases decreased, with cell disintegration and structural loss. In the experimental group, the structures of the lungs and livers remained essentially normal. (**Figure 7C**).

Next, we performed immunofluorescence staining on the livers to observe the relative expression levels of CENPN, p-STAT3, and USP37. The results revealed that the fluorescence intensities of the three proteins in the shCENPN group were significantly lower than those in the control group (**Figure 7D**). We conducted western blotting experiments on liver homogenates from the mice to further detect the expression levels of the related proteins. Compared with those in the control group, the expression levels of CENPN, p-STAT3, USP37, and Snail in the shCENPN group were significantly lower, which was consistent with the results of the *in vitro* experiments (**Figure 7E**).

### 3.8 CENPN/STAT3/USP37 is strongly associated with metastasis for patients with NPC

Nasopharyngeal carcinoma is a subtype of head and neck squamous cell carcinoma (HNSCC). Using the GEPIA tool to analyze data from HNSCC patients in TCGA database, we found that the CENPN expression level was significantly higher in the tumor group than in the control group (**Supplementary Figure S3A**). An expression analysis of four datasets in the GEO database revealed that CENPN expression was significantly higher in NPC patients than in the controls (**Supplementary Figure S3B**). The survival analysis indicated that HNSCC patients with high CENPN expression had poorer survival prognoses (**Supplementary Figure S3C**).

**Figure 8:**
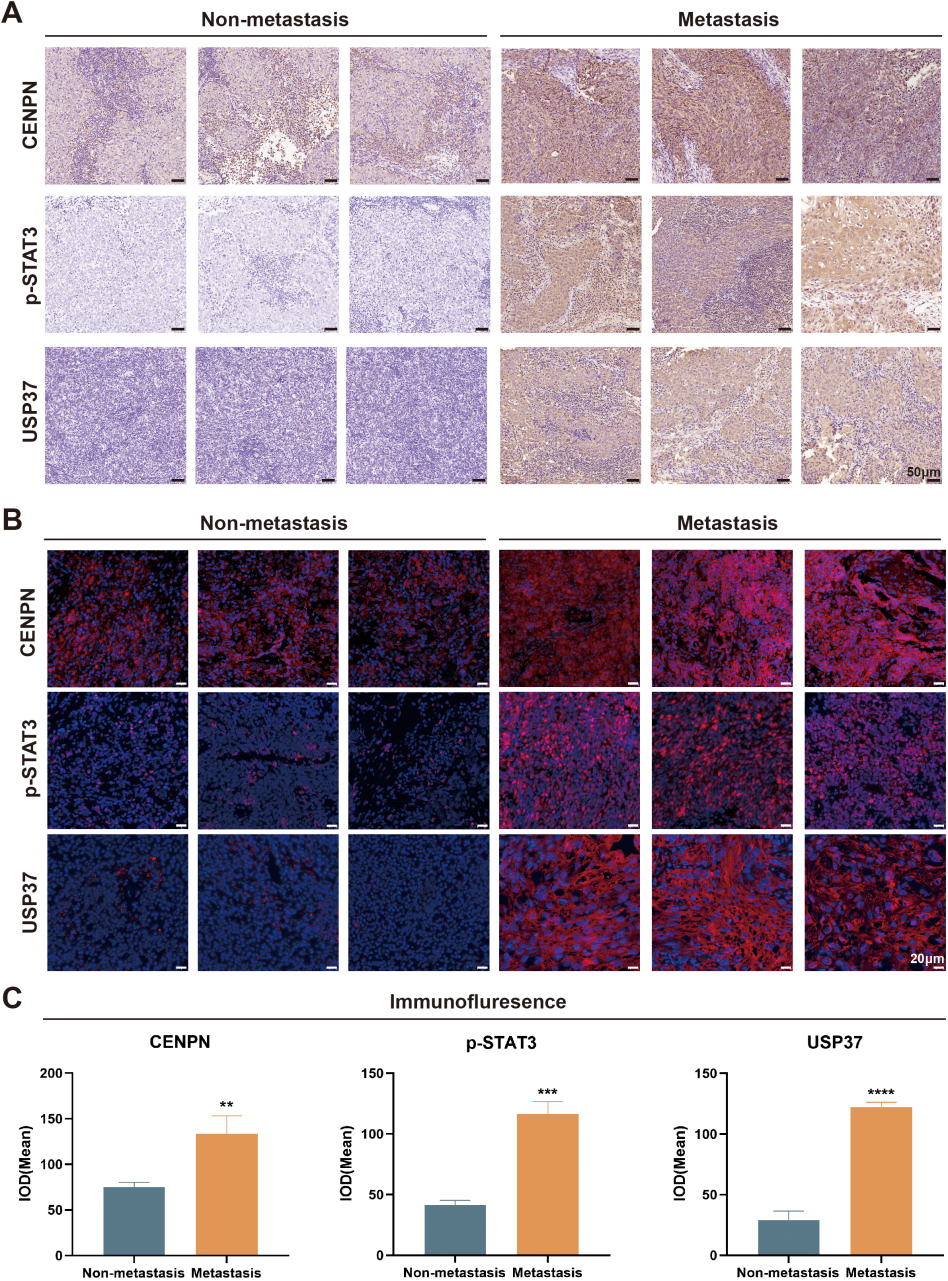
The CENPN/STAT3/USP37 axis is closely associated with nasopharyngeal carcinoma metastasis. **(A)** Representative image of immunohistochemical staining in the metastatic and nonmetastatic nasopharyngeal carcinoma (200×). **(B)** Representative tissue immunofluorescence staining results in the metastatic and nonmetastatic nasopharyngeal carcinoma (400×). **(C)** The statistical analysis of the immunofluorescence staining results. ** p<0.01, *** p<0.001 and **** p<0.0001.

We selected surgical samples from patients with metastatic and nonmetastatic NPC for immunohistochemical (IHC) and immunofluorescence (IF) staining to verify the expression of CENPN in metastatic NPC and the relationship between the CENPN/STAT3/USP37 axis and NPC metastasis. The IHC results revealed that the expression levels of CENPN, p-STAT3, and USP37 were significantly higher in the metastatic group than in the nonmetastatic group (**Figure 8A**). The statistical analyses are shown in **Supplementary Figure S4**. The IF staining scores were consistent with the IHC results (**Figures 8B, C**). The expression level of CENPN was positively correlated with the expression level of p-STAT3 and USP37 (**Supplementary Figure S5**).

## 4 Discussion

The CENPN gene encodes centromere protein N, which plays a crucial role in cell division and acts as an oncogene involved in the progression of various types of tumors. Our previous study revealed that CENPN is closely related to the malignant biological behaviors of nasopharyngeal carcinoma (NPC) cells, such as glucose metabolism, autophagy, and radioresistance (16, 32, 35). In other types of tumors, studies have shown that CENPN can also promote the metastasis of gliomas, breast cancer and other cancers (17, 18). However, the role of CENPN in the invasion and metastasis of NPC and its potential molecular mechanisms currently remain unclear.

Tumor invasion and metastasis are key characteristics of cancer progression and the main causes of cancer-related death (36, 37). The epithelial–mesenchymal transition (EMT) imparts invasive phenotypes to tumor cells and is a critical process in tumor metastasis. At the molecular level, the EMT is characterized by the downregulation of epithelial proteins (such as the adhesion junction protein E-cadherin) and the acquisition of mesenchymal markers (such as the Snail and vimentin proteins) (38). In this study, we found that knockdown of CENPN in NPC cells resulted in decreased expression of Snail and vimentin and increased expression of E-cadherin, significantly reducing the invasion and metastasis capabilities. Conversely, overexpression of CENPN markedly increased Snail and vimentin expression, decreased E-cadherin expression, and significantly enhanced the invasion and metastasis capabilities. Consistent with our results, Wu et al. reported that CENPN knockdown significantly inhibited the invasion and metastasis of glioma cells (17). However, the specific molecular mechanisms by which CENPN regulates tumor cell invasion and metastasis have not yet been clearly reported.

Transcriptome sequencing, a type of high-throughput sequencing technology, plays a significant role in cancer research. It can effectively address the challenges posed by the complexity of tumor genomes, providing important information for the molecular mechanisms and diagnosis and treatment of tumors (39). We performed transcriptome sequencing on CENPN-knockdown 5-8F cells and control cells to identify the downstream genes affected by CENPN that influence NPC metastasis. The results revealed a significant decrease in USP37 expression following CENPN knockdown. Many studies have confirmed that USP37, a stabilizer of oncogenic proteins, can promote tumorigenesis and progression (40). He et al. highlighted the prominent role of USP37 in promoting tumor metastasis (26, 41). As a member of the deubiquitinase family, USP37 can deubiquitinate the Snail protein, protecting it from proteasomal degradation (27, 30, 31). The Snail protein regulates the expression of EMT-related proteins, and its increased stability significantly promotes the EMT process (29, 42). Therefore, we speculated that CENPN may regulate NPC metastasis through the USP37-Snail pathway. We used western blotting and qRT-PCR to verify this hypothesis and found that USP37 expression was significantly reduced in NPC cells after CENPN knockdown. Overexpression of USP37 in CENPN-knockdown NPC cells led to a notable recovery of the cells’ invasion and metastasis capabilities and Snail protein expression levels compared to cells with CENPN knockdown alone. These findings confirm that CENPN influences NPC invasion and metastasis by regulating USP37 expression.

**Figure 9:**
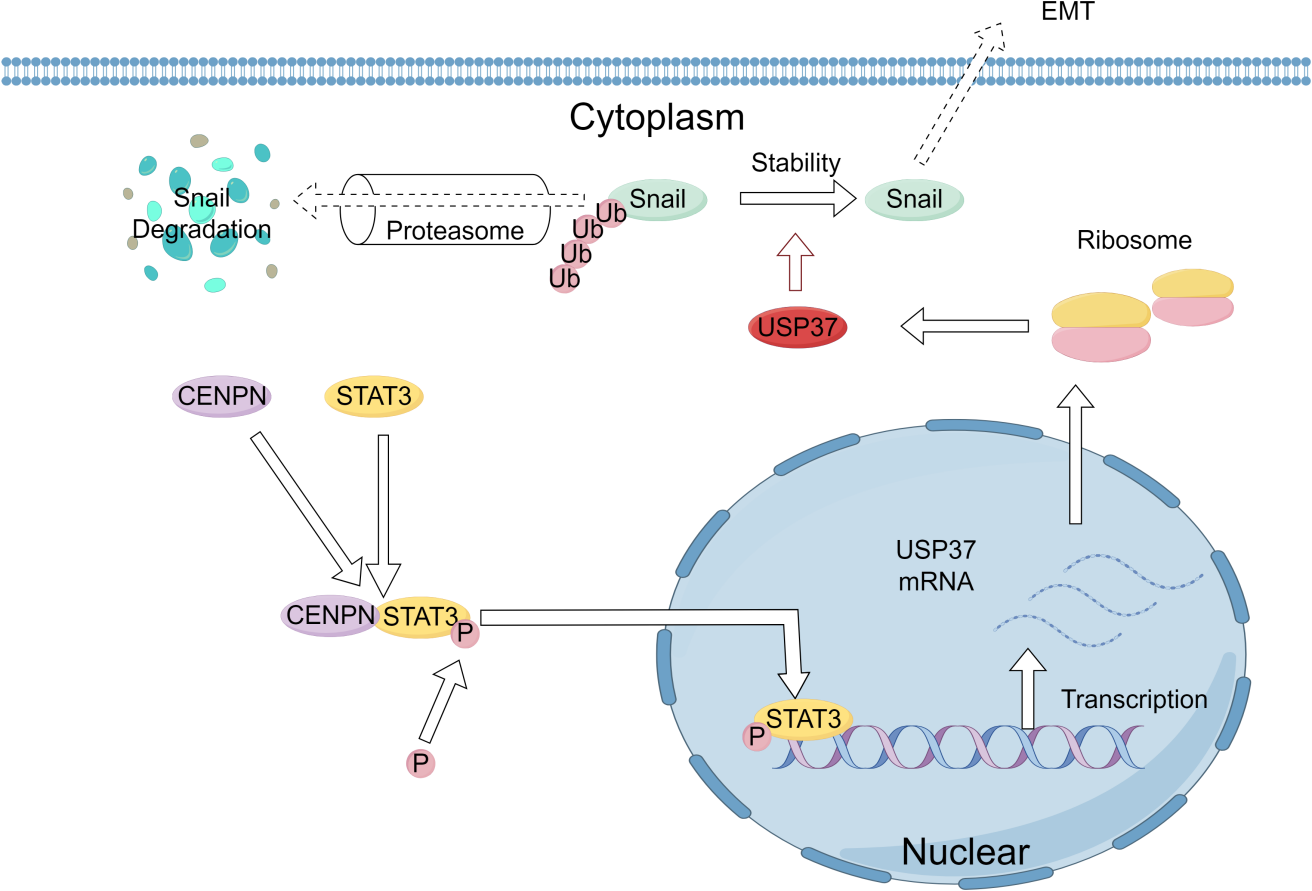
Schematic of the molecular mechanism by which CENPN enhances the proliferation and invasion of nasopharyngeal carcinoma. Elevated CENPN expression promotes STAT3 phosphorylation and nuclear translocation, activating USP37 expression to protect the Snail protein from proteasomal degradation and thereby promoting nasopharyngeal carcinoma metastasis.

Although CENPN expression is closely related to USP37 expression, CENPN cannot directly regulate the expression of USP37. Therefore, we predicted transcription factors and their binding sites using databases such as JASPAR and UCSC and speculated that STAT3 might be a transcription factor for USP37. Previous studies have reported that STAT3, an oncogenic factor, can promote the proliferation and survival of tumor cells and increase their invasion and migration abilities (25, 43–45). STAT3 is activated by phosphorylation in the cytoplasm and then enters the nucleus through nuclear pores to bind to specific sequences of target genes, thereby regulating gene transcription (21). Our study revealed that the expression of CENPN can concomitantly alter the phosphorylation level of STAT3, the expression level of USP37, and the EMT phenotype. Further research revealed a direct interaction between the CENPN and STAT3 proteins. The binding of CENPN to STAT3 promotes the phosphorylation and nuclear translocation of STAT3. Once p-STAT3 enters the nucleus, it binds to the USP37 promoter region, transcriptionally activating the expression of USP37. Therefore, this study confirms that CENPN promotes the invasion and metastasis of NPC cells by regulating the STAT3-USP37 axis. The detailed mechanism is illustrated in **Figure 9**.

In recent years, the application of small-molecule inhibitors in cancer therapy has made significant progress and has gradually become an important method of modern cancer treatment. These drugs can target specific molecular pathways or proteins, blocking the growth and spread of cancer cells (46). Our previous research showed that the IRF2-CENPN-AKT axis promotes NPC cell proliferation and resistance to apoptosis by enhancing aerobic glycolysis. CENPN can inhibit autophagy and enhance chemotherapy resistance in NPC cells by downregulating CREB-VAMP8 expression. Additionally, it can enhance radioresistance in NPC cells through the AKT/mTOR signaling pathway (16, 32, 35). The results of this study confirmed that CENPN promotes the invasion and metastasis of NPC cells by regulating STAT3-USP37 expression. Taken together, these findings indicate that CENPN promotes the occurrence and development of NPC in multiple ways, suggesting that specific small-molecule drugs targeting CENPN are promising for NPC therapy.

Moreover, this study has certain limitations. Since most NPC patients seek medical attention at mid-to-late stages, often with lymph node or distant metastases, our study collected clinical specimens from 40 NPC patients, only 10 of whom had nonmetastatic NPC. In the future, more patients, especially patients with nonmetastatic disease, should be included to further verify our experimental results. Additionally, in tissue specimens, CENPN expression was significantly higher in NPC tissues than in control tissues. Therefore, in our mouse experiments, we observed only the effect of CENPN knockdown on NPC cell metastasis *in vivo*. In the future, conditions permitting, we can use NPC cells with upregulated CENPN expression for *in vivo* experiments to further validate our research results. While this study elucidates the functional role of the CENPN/STAT3/USP37 axis in driving NPC progression at the molecular and genetic level, cancer’s multifaceted nature—spanning genetic, cellular, microenvironmental, and systemic dynamics—demands interdisciplinary exploration beyond molecular mechanisms alone. Future work will therefore integrate tumor microenvironment dynamics, particularly immune evasion mechanisms and stromal remodeling processes, to enhance the clinical translatability of these findings and pioneer multimodal therapeutic interventions.

In conclusion, our research indicates that CENPN promotes the invasion and metastasis of NPC by promoting the phosphorylation and nuclear translocation of STAT3, which in turn regulates USP37 transcription. The CENPN/STAT3/USP37 axis has the potential to become a new therapeutic target for NPC.

## Data Availability

The original contributions presented in the study are publicly available. This data can be found here: [https://figshare.com/ DOI: 10.6084/m9.figshare.29052638].
