# Supplementary material for: The CENPN/STAT3/USP37 signaling axis promotes invasion, migration and metastasis in nasopharyngeal carcinoma
Source: Front Oncol. 2025 May 19;15:1536574. doi: 10.3389/fonc.2025.1536574 (PMC12127386; doi:10.3389/fonc.2025.1536574)
Supplement: Supplementary file 1 [file DataSheet1.docx]

Supplementary Material

# Supplementary Tables

## Supplementary Table 1. The website addresses of datasets

| Dataset | Web Link |
| --- | --- |
| GEO | <https://www.ncbi.nlm.nih.gov/geo/> |
| STRING | <http://string.embl.de/> |
| Kaplan–Meier plotter | <http://kmplot.com/analysis/> |
| GEPIA  GENE | <http://gepia.cancer-pku.cn/>  https://www.ncbi.nlm.nih.gov/gene/ |
| GeneCards  PROMO | <http://www.genecards.org/>  http://alggen.lsi.upc.es/ |
| JASPER | http://jaspar.genereg.net/ |
| GEPIA  KEGG  GO | <http://gepia.cancer-pku.cn/>  <http://www.genome.jp/kegg/>  <http://www.geneontology.org> |

## Supplementary Table 2. The references for all antibodies used

| Antibodies | Company | Number | Country | Applications |
| --- | --- | --- | --- | --- |
| CENP-N | Absin^a^ | abs106804 | CHN | WB, IF, IHC |
| CENP-N | Novus^b^ | H00055839-PW1 | USA | IP |
| USP37 | Abcam^c^ | ab72199 | UK | IF, IHC, WB |
| STAT3 | Proteintech^d^ | 10253-2-AP | USA | WB |
| p-STAT3  (Tyr705) | Affinity Biosciences^e^ | AF3293 | USA | WB, IF, IHC, IP |
| E-Cadherin | Proteintech^d^ | 20874-1-AP | USA | WB, IF |
| Vimentin | Proteintech^d^ | 10366-1-AP | USA | WB, IF |
| Snail | Proteintech^d^ | 13099-1-AP | USA | WB |
| β-tubulin | Proteintech^d^ | 10068-1-AP | USA | WB |
| Secondary Antibody | Proteintech^b^ | SA00001-2 | USA | WB |

Note: ^a^<https://www.absin.cn/>, ^b^<https://www.novusbio.com/>, ^c^<https://www.abcam.com/>, ^d^<https://www.ptgcn.com/>, ^e^<https://www.affbiotech.cn/>, WB: western blot analysis, IP: immunoprecipitation, IHC: Immunohistochemical staining analysis, IF: immunofluorescence.

## Supplementary Table 3. List of upregulated and downregulated genes identified from the transcriptomic analysis following CENPN knockdown

| **Upregulated genes** | **Downregulated genes** |
| --- | --- |
| PDHB | ENO1P3 |
| KRTCAP2 | CCND2 |
| ARPC2 | MYCBP2 |
| VAMP8 | MKI67 |
| TWF2 | BCLAF1 |
| RPL26 | CDK12 |
| OAZ1 | BCLAF1P2 |
| CD99 | CCNDBP1 |
| RPS27 | CDK6 |
| ITM2C | CDK14 |
| SERPINE2 | CENPN |
| HSD17B10 | SPRY1 |
| AP2S1 | USP37 |
| COPZ1 | NBEAL1 |
| RPL31 | ARMC5 |
| RAB11FIP5 | GAN |
| EBP | NFIA |
| MAD2L2 | ABCA11P |
| PKX | LRIG2 |
| Sp8 | HECTD1 |
| CASP14 | PAXBP1 |
| AC005062.1 | DHX8 |
| EMC6 | EP300 |
| PSMB9 | MAN1A2 |
| POMT2 | AP000919.1 |
| 8-MAR | SCARNA18 |
| CATSPER1 | SNORA10 |
| PDZD4 | WDR66 |
| DHRS13 | MIER1 |
| DYSF | CSNK1G3 |
| HS3ST1 | AL080251.1 |
| RGPD6 | PLCG1-AS1 |
| NR2F1 | AC007919.2 |
| PXK | RNU4-54P |
| SNAI1 | SYNJ1 |
| SEPT5 | AC244453.4 |
| MMP25-AS1 | CCDC142 |
| ZNF251 | ZNF33BP1 |
| AC009779.2 | AC098591.2 |
| LRCH4 | MORF4L1P3 |
| CGRRF1 | AC012676.1 |
| MSRB3 | RPL21P8 |
| ARID3A | AL022238.1 |
| TUBG2 | AL080317.2 |
| LINC00205 | PLEKHB1 |
| CACNA1H | AL121989.1 |
| FAM171A2 | AC099811.6 |
| LINC01204 | KSR1 |
| RTKN2 | AL391262.1 |
| ZFAND4 | Metazoa_SRP |
| KIF21B | AC027020.2 |
| SLC1A6 | MCPH1 |
| SMIM2-AS1 | SNORD15A |
| TRMT112P6 | FUT2 |
| DTX2P1 | PQLC2 |
| BET1L | AC135050.7 |
| GDF15 | SNORA22 |
| AC000123.2 | AC040160.2 |
| DMRTA1 | MT-TT |
| PSMG3-AS1 | AC092903.2 |
| AC109454.3 | AC114402.2 |
| LINC01556 | AC253536.6 |
| NPAS1 | AC007608.4 |
| SCAMP5 | HIST1H1E |
| LINC02542 |  |
| AURKC |  |
| AL118558.3 |  |
| FTH1P8 |  |
| RPS28 |  |
| PSMB4 |  |
| TMEM189 |  |
| YKT6 |  |
| GABARAPL2 |  |
| CNOT8 |  |
| VASP |  |
| AC009245.1 |  |
| IMP3 |  |
| HADHA |  |
| NQO2 |  |
| MYL12A |  |
| GALNT2 |  |
| EPAS1 |  |
| SERINC1 |  |

# Supplementary Figures

## Supplementary Figure 1


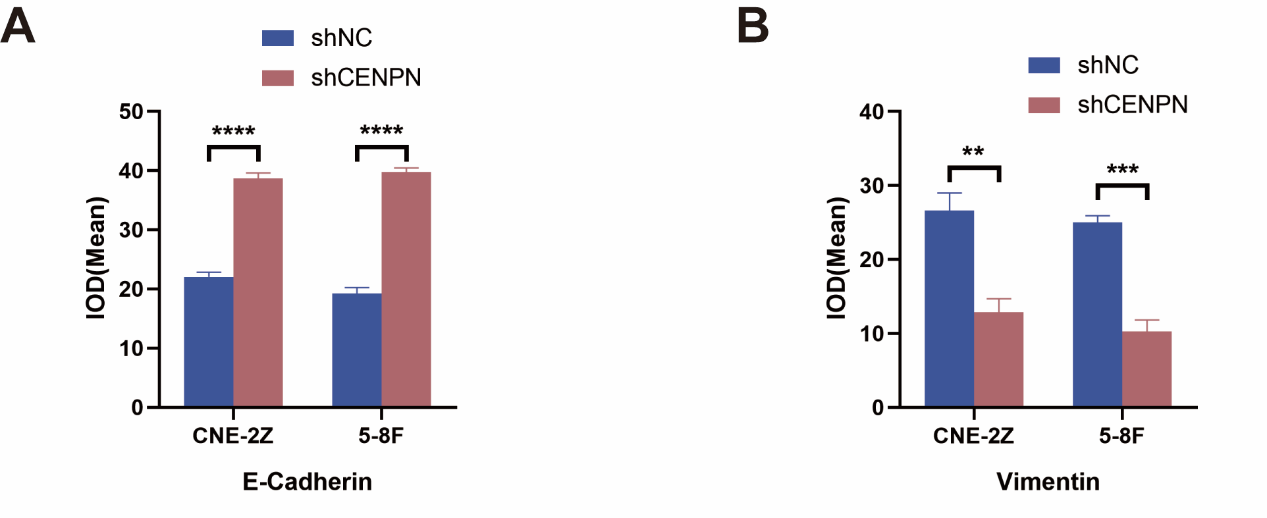


**Supplementary Figure 1.** Changes of relative expression levels of E-cadherin and vimentin in CNE-2Z and 5-8F cell lines after CENPN knockdown. ** p<0.01, *** p<0.001 and **** p<0.0001.

## Supplementary Figure 2


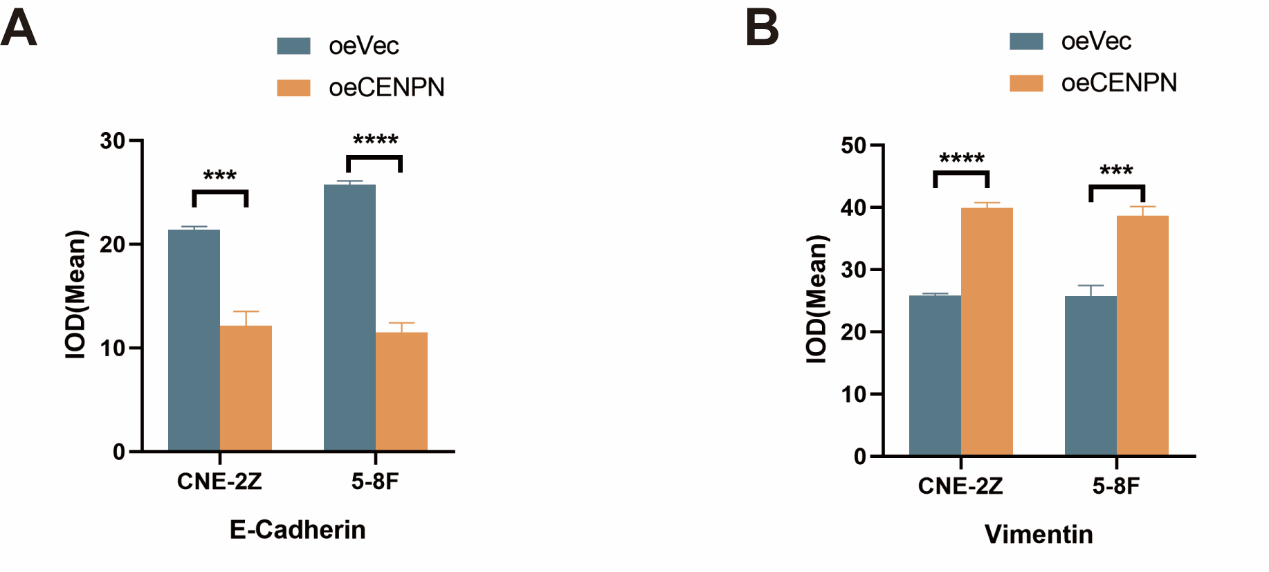


**Supplementary Figure 2.** Changes of relative expression levels of E-cadherin and vimentin in CNE-2Z and 5-8F cell lines after CENPN overexpression. *** p<0.001 and **** p<0.0001.

## Supplementary Figure 3


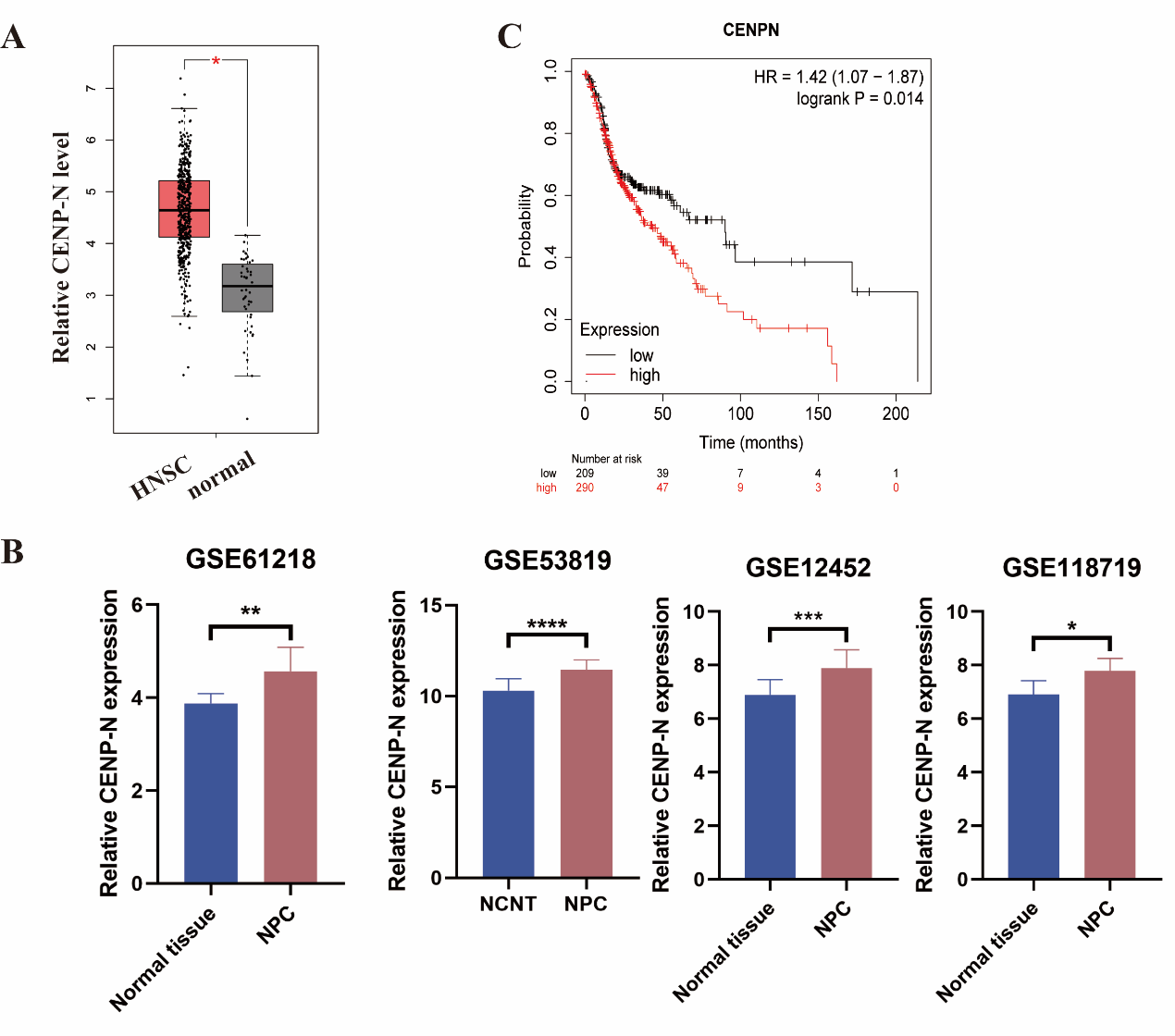


**Supplementary Figure 3.** CENPN expression is closely related to HNSC occurrence and poor prognosis. (A) Relative expression level of CENPN in HNSC and control group in TCGA database.
(B) Relative expression of CENPN in NPC tissues and control group in GSE61218, GSE53819, GSE12452, GSE118719 datasets. (C) Survival curves of HNSC patients in the high and low CENPN expression groups. * p<0.05, ** p<0.01, *** p<0.001 and **** p<0.0001.

## Supplementary Figure 4


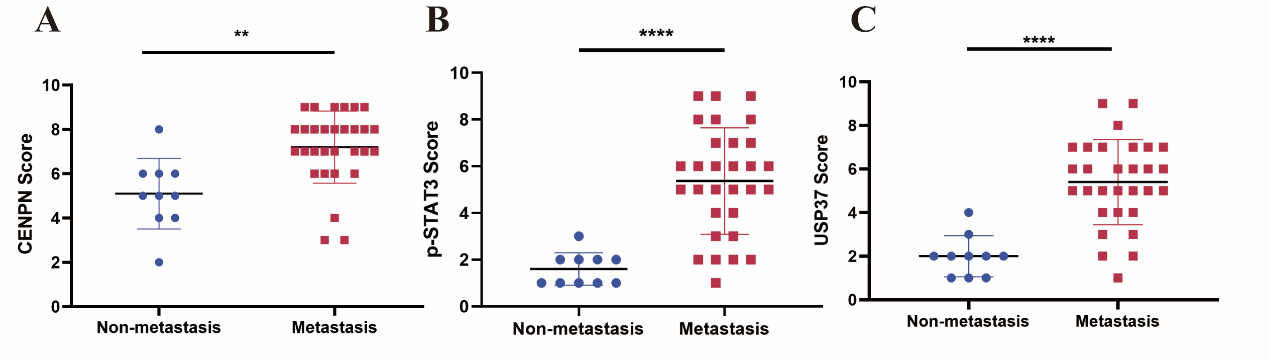


**Supplementary Figure 4.** Immunohistochemical scores of CENPN, p-STAT3 and USP37 in 40 nasopharyngeal carcinoma patients. ** p<0.01 and **** p<0.0001.

## Supplementary Figure 5


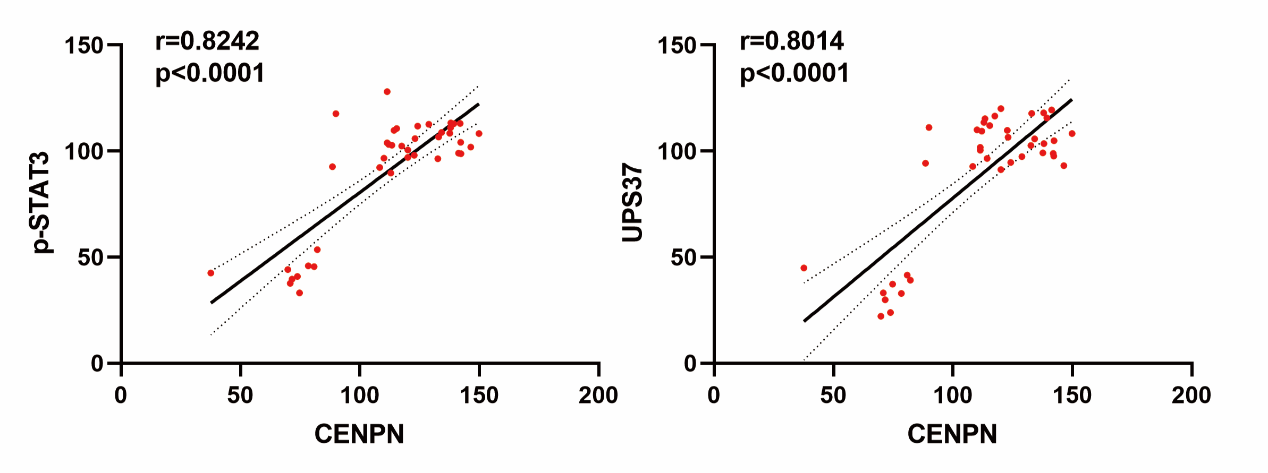


**Supplementary Figure 5.** Correlation analysis of CENP-N, p-STAT3 and USP37 protein expression levels in nasopharyngeal carcinoma tissues.
